# Hot electron-induced reduction of small molecules on photorecycling metal surfaces

**DOI:** 10.1038/ncomms8570

**Published:** 2015-07-03

**Authors:** Wei Xie, Sebastian Schlücker

**Affiliations:** 1 Physical Chemistry I, Faculty of Chemistry and Center for Nanointegration Duisburg-Essen (CENIDE), University of Duisburg-Essen, Universitätsstr. 5, Essen 45141, Germany

## Abstract

Noble metals are important photocatalysts due to their ability to convert light into chemical energy. Hot electrons, generated via the non-radiative decay of localized surface plasmons, can be transferred to reactants on the metal surface. Unfortunately, the number of hot electrons per molecule is limited due to charge–carrier recombination. In addition to the reduction half-reaction with hot electrons, also the corresponding oxidation counter-half-reaction must take place since otherwise the overall redox reaction cannot proceed. Here we report on the conceptual importance of promoting the oxidation counter-half-reaction in plasmon-mediated catalysis by photorecycling in order to overcome this general limitation. A six-electron photocatalytic reaction occurs even in the absence of conventional chemical reducing agents due to the photoinduced recycling of Ag atoms from hot holes in the oxidation half-reaction. This concept of multi-electron, counter-half-reaction-promoted photocatalysis provides exciting new opportunities for driving efficient light-to-energy conversion processes.

## Abstract (web summary)

Hot electrons are generated when energy is transferred from an incoming photon, enabling an electron from a metal surface to become mobile. Here, the authors irradiate plasmonically active silver core-satellite superstructures and use the hot electrons to effect chemical reactions via photorecycling.

## Graphical abstract

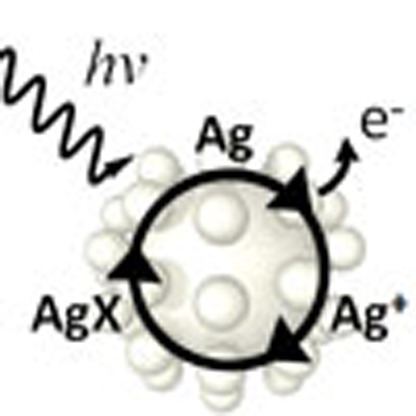

Noble metal nanoparticles are nanoscale sources of light, heat and electrons^1^. This can be exploited in numerous applications such as plasmon-assisted optical microscopy and spectroscopy (light)^2^, photothermal therapy (heat)^3^ and plasmon-driven redox reactions in chemistry (electrons)^4^,^5^,^6^.

Upon resonant illumination with light in the visible range, the free electron gas of a metal nanoparticle is externally driven to perform oscillations at its plasma eigenfrequency. This resonant electronic excitation is called localized surface plasmon. There are two fundamentally different decay channels for localized surface plasmons: a radiative channel, in which the nanoparticle acts as a nanoantenna (scattering), and a non-radiative channel (absorption). The absorption of photons may lead to the generation of heat by electron–phonon coupling or to the generation of charge carriers by the excitation of electron–hole pairs^7^. In the latter case, the generated high-energy electrons can be transferred from the surface of the metal nanoparticle to an adjacent electron acceptor such as a semiconductor or a molecule^8^,^9^,^10^,^11^.

The radiative channel is the physical basis of plasmon-assisted light scattering. Chemically relevant information on molecules adsorbed on the surface of metal nanoparticles can be obtained via surface-enhanced Raman scattering (SERS)^12^,^13^,^14^, which inherits the high molecular specificity of Raman spectroscopy, but with signal levels that are orders of magnitude higher^15^,^16^. In heterogeneous catalysis, SERS has been used as a surface-selective tool for label-free monitoring of chemical reactions^17^,^18^. Proof-of-concept studies were focused on monitoring the metal nanoparticle-catalyzed reduction of aromatic nitro compounds by hydride reagents^19^,^20^,^21^. So far, Ag nanoparticles have not been able to catalyse this reaction under otherwise same conditions.

Interestingly, we discovered that even in the absence of hydride reagents, this six-electron reduction reaction can take place on the surface of Ag nanoparticles in the presence of protons and halide ions. Our accidental observation has only been possible by employing highly plasmonically active Ag superstructures for label-free monitoring of the reaction by *in situ* SERS spectroscopy since otherwise the product could not have been identified. Central elements in this plasmon-mediated reduction reaction are high-energy hot electrons and halide ions. The hot electrons are transferred to the adsorbed molecules in the reduction half-reaction, while the hot holes are responsible for the oxidation counter-half-reaction. Halide ions are a key component in this redox reaction since they form photosensitive silver halides. The photoinduced dissociation of the silver halides acts as the oxidation half-reaction to compensate the hot holes and facilitate the electron transfer to the adsorbate. Importantly, it this recycling of surface silver atoms which drives the overall redox reaction.

## Results

### Core–satellite Ag superstructures

**Figure 1:**
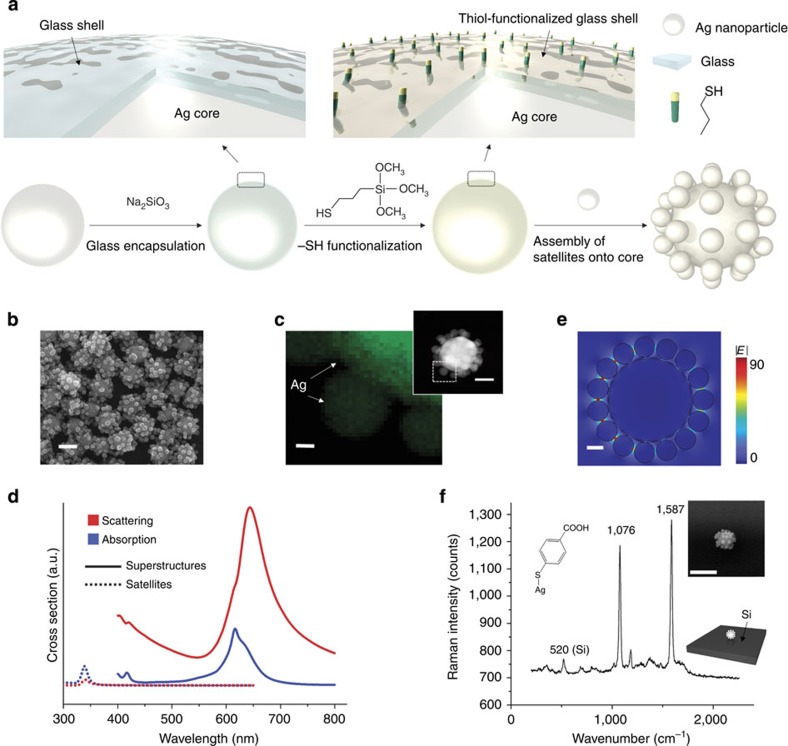
Synthesis and characterization of highly plasmonically active Ag core–satellite superstructures. (**a**) Scheme of the synthesis: 100 nm Ag cores are encapsulated with an ultrathin glass shell and functionalized with thiol groups. Ag satellite nanoparticles (25 nm) are assembled onto the shell-isolated Ag core. (**b**) SEM image of the Ag superstructures (scale bar, 100 nm). (**c**) EDS element map (Ag) of the superstructure (scale bar, 10 nm) and the corresponding STEM image (scale bar, 50 nm). (**d**) Calculated absorption and scattering cross-sections of a 25 nm Ag satellite compared with the Ag superstructure. The cross-sections in both channels, absorption for efficient generation of hot electron–hole pairs and scattering for chemical monitoring with molecular specificity by SERS, are much larger in the superstructure. (**e**) Finite element method simulation of the incident electric field amplitude |*E*| distribution on resonant excitation at 632.8 nm (scale bar, 20 nm). (**f**) SERS spectrum of 4-MBA from a single Ag superstructure on a Si wafer shown in the SEM image (top right; scale bar, 200 nm). The strong SERS peaks of 4-MBA at 1,076 and 1,587 cm^−1^ (laser power 0.6 mW and intergration time 36.5 ms; first-order phonon peak of silicon at ∼520 cm^−1^) demonstrate the single-particle SERS activity of the Ag superstructures.

Figure 1a shows a multiple-step method for the synthesis of core–satellite Ag superstructures. Silver nanoparticle cores with a diameter of ∼100 nm are first encapsulated by an ultrathin silica shell. The silica surface is then functionalized with thiol groups. Silver nanoparticle satellites (∼25 nm) are assembled onto the large core via Ag–S bonds. Silver superstructures with dozens of satellites can be synthesized in large scale (Fig. 1b,c).

High plasmonic activity is required for the generation of hot electrons for redox chemistry (non-radiative plasmon decay) and for SERS reaction monitoring (radiative plasmon decay). Although silver is one of the most plasmonically active metals, Ag nanoparticle monomers do not have sufficiently high scattering cross-sections for SERS monitoring (Supplementary Figs 1–3 and Supplementary Note 1). In contrast, our rationally designed core/satellite superstructures (Supplementary Fig. 4) exhibit significantly larger absorption and scattering cross-sections (Fig. 1d). Computer simulations predict a local electric field enhancement of |*E*| ∼90 upon resonant excitation of the Ag superstructure (Fig. 1e and Supplementary Fig. 5). This corresponds to a SERS enhancement factor^15^ (EF) of |*E*|^4^ ∼6.62 × 10^7^. Experimentally, the very high plasmonic activity of the Ag superstructures is demonstrated in single-particle SERS experiments (Supplementary Fig. 6). Figure 1f shows the enhanced Raman spectrum of 4-mercaptobenzoic acid (4-MBA) on a single Ag superstructure on a silicon wafer. The Raman intensity of dominant peaks from 4-MBA (1,076 and 1,587 cm^−1^) is about 10 times stronger than the first-order phonon peak from the silicon substrate at ∼520 cm^−1^. This single-particle SERS activity enables quantitative and label-free reaction monitoring.

### Hot electron reduction

**Figure 2:**
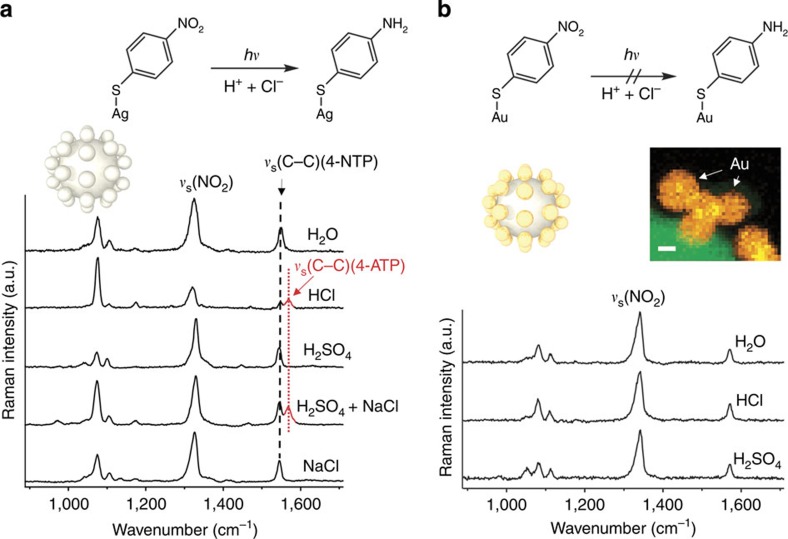
Hot electron-induced reduction from 4-NTP to 4-ATP on Ag versus on Au surfaces. (**a**) SERS spectra of 4-NTP on the surface of plasmonic Ag superstructures in different environments. 4-ATP, the product of the hot electron-induced reaction, can be detected (the C–C and C–S stretching bands of 4-ATP appear at ∼1,590 and ∼1,080 cm^−1^, respectively) only in the presence of both H^+^ and Cl^−^. (**b**) SERS control experiments using the ∼25 nm Au satellites of the superstructure. The inset (middle right) shows the element mapping of both Au and Ag (scale bar, 10 nm). It is important to mention that the 4-NTP educt molecules can only adsorb on the satellite particle surface because the Ag core is isolated by an ultrathin glass shell. No signal from 4-ATP was detected on the Au surface, indicating that the reduction cannot proceed on Au nanoparticles even in the presence of hot electrons. The hot holes remaining on the Au surface limit the number of hot electrons that can be transferred to the 4-NTP molecules. See Fig. 3 for proposed reaction mechanism.

The Ag superstructures were coated with a self-assembled monolayer (SAM) of 4-nitrothiophenol (4-NTP). The reduction of the educt 4-NTP to the product 4-aminothiophenol (4-ATP) by sodium borohydride has been used as a model reaction in previous proof-of-concept studies to test the catalytic activity of noble metal nanoparticles such as Au, Pt and Pd^19^,^20^,^21^,^22^,^23^. In contrast, Ag nanoparticles cannot catalyse this hydride (H^−^) reduction reaction (Supplementary Figs 7 and 8 and Supplementary Note 2). Surprisingly, we detected the SERS signal of the reaction product 4-ATP when the Ag core/satellite superstructures were suspended in aqueous HCl (Fig. 2a). This unexpected finding is exciting since it demonstrates that even in the absence of a chemical hydride agent, the reduction on much cheaper Ag surfaces is now possible. Formally, the hydride equivalent in this photocatalytic reaction is provided by a proton and two electrons: H^−^=H^+^+2e^−^. In control experiments we tested other acids such as H_2_SO_4_. However, in this case only the SERS signal of the educt 4-NTP and no contributions from the product 4-ATP were detected, indicating that the reduction did not occur. This led us to the hypothesis that not only acidic conditions, but also the counter anion is relevant. We tested this by adding NaCl to the Ag superstructures suspended in H_2_SO_4_ and indeed the SERS signal of the product 4-ATP was detected. In a negative control experiment, we added only aqueous NaCl solution, that is, without acid, to the Ag superstructures. As expected, no SERS signal of the product 4-ATP was observed. We therefore concluded that both protons and chloride anions are required for this photocatalytic reduction. While protons are obviously needed as the hydrogen source in this reaction, we initially did not have an explanation for the role of the chloride anions.

The generation of hot electrons is in principle possible with all plasmonic materials upon resonant excitation by light. Tuning the laser excitation wavelength away from *λ*_max_ of the plasmon peak leads to lower reduction activity (Supplementary Fig. 9 and Supplementary Note 3). We therefore assumed that this unexpected photocatalytic route on Ag, involving hot electrons but no chemical reducing agent, may also occur on Au surfaces. In order to test this, we synthesized hybrid Ag core–Au satellite superstructures since the Au satellites might donate their hot electrons to the SAM on their surface. It is important to mention that this is only possible for the satellites (Au), but not for the core (Ag): the large Ag core is isolated by an inert ultrathin glass as a dielectric spacer, which prevents the chemisorption of molecules onto the surface of the core as well as the transfer of hot electrons to them^24^. To our surprise, no reduction reaction was observed for the Ag core–Au satellite superstructures (Supplementary Fig. 10), neither in aqueous HCl nor aqueous H_2_SO_4_ (Fig. 2b and Supplementary Note 4). This finding indicates that not only the plasmonic but also the chemical properties of the metal (Supplementary Figs 11 and 12), in particular Ag, are relevant for driving this photocatalytic reaction. In order to find out whether a chloride-specific effect is present, we also tested other halide anions.

### Counter-half-reaction

**Figure 3:**
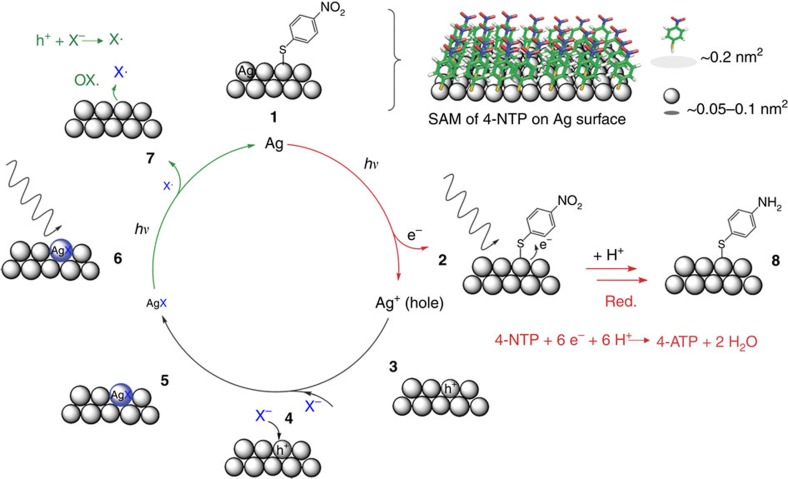
Counter-half-reaction-promoted hot electron reduction. Schematic illustration of the excitation of hot electrons for the reduction of 4-NTP to 4-ATP. Halide anions compensate the hot holes via the oxidation counter-half-reaction, thereby recycling the plasmonic Ag surface. When a SAM of 4-NTP molecules covers the Ag surface (**1**), each molecule occupies about 2–4 surface Ag atoms (see top right of the figure). However, six electrons per 4-NTP molecule (**2**) are required to complete the reduction to 4-ATP (**8**). Thus, the Ag superstructure (**3**) has to gain electrons from outside via the oxidation half-reaction (labelled with green in the reaction circle). Due to the very low solubility of the corresponding silver halides, the halide anions have a very strong affinity to the hot holes (Ag^+^) (**4**) and lead to the formation of photosensitive AgX (**5**), which undergoes a photodissociation on the Ag surface (**6**). Thus, the oxidation half-reaction (**7**) is established due to this formation and subsequent decomposition of the photoactive AgX. In this case the hot holes are compensated and thereby recycle the Ag surface for the hot electron-induced reduction half-reaction (labelled in red).

Silver chloride has a very low solubility in water (*K*_SP_=1.77 × 10^−10^ M^2^ at room temperature) and, more importantly, it is a photosensitive salt that has been used in photography since the early 19th century. The proposed reaction mechanism of this photocatalytic reaction, which is in agreement with all experimental findings, is shown in Fig. 3. First, hot electron–hole pairs (e^−^/h^+^) are generated on the Ag superstructure upon resonant light excitation:


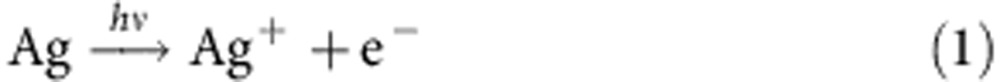


The hot electrons can be transferred to the molecules chemisorbed on the Ag surface. In the absence of chloride anions the number of the hot electrons is not sufficient for driving the reaction due to the high charge–carrier recombination rate. In the presence of Cl^−^ the hot holes (h^+^=Ag^+^) combine with Cl^−^ ions and form photosensitive AgCl on the Ag surface, which is then decomposed via photodissociation:


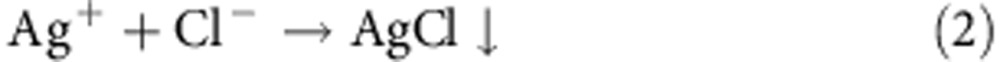



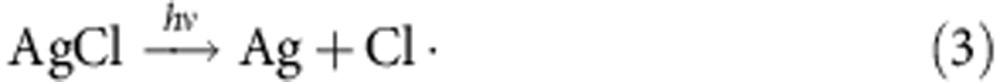


In this case the oxidation half-reaction is the electron transfer from Cl^−^ to the Ag catalyst and more hot electrons can be provided for the reduction half-reaction. The Cl^−^ ions are therefore required for recycling Ag atoms as the electron donors from the corresponding hot holes (h^+^=Ag^+^). This photorecycling is a necessary condition since overall six electrons are formally needed to drive this reduction reaction:


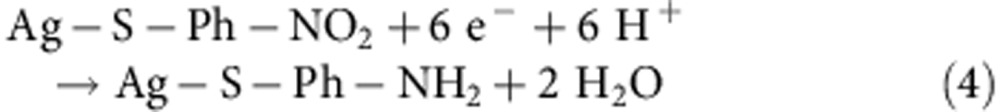


A single 4-NTP molecule in the SAM occupies about 0.2 nm^2^ Ag surface area^25^, where only 2–4 Ag atoms are present (Fig. 3). Because of the fast charge–carrier recombination it is not possible that the Ag surface can provide enough hot electrons for the entire reduction alone, that is, without recruiting additional electrons from the environment.

**Figure 4:**
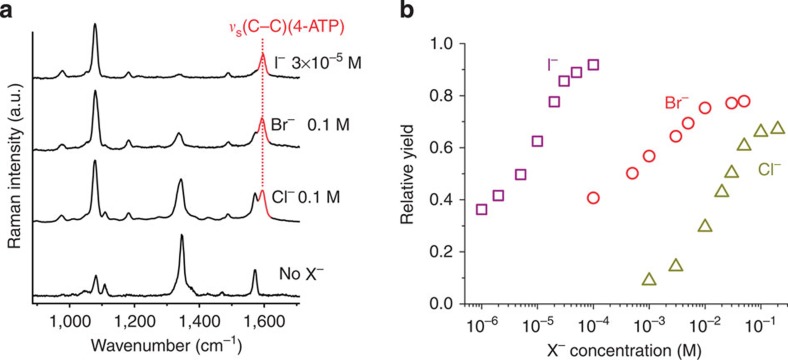
Hot electron-induced reduction in the presence of halide anions. (**a**) SERS spectra of the reactions on plasmonic Ag superstructures using different halide anions (Cl^−^, Br^−^ and I^−^) to compensate hot holes (h^+^=Ag^+^). Iodide shows the strongest activity for the reaction even at very low concentrations. (**b**) Relative contribution of the product (4-ATP) to the SERS spectra from the reaction suspension as a function of halide anion concentration. The higher activity of I^−^ is because the corresponding silver halide has a lower solubility *K*_sp_ (AgI=8.52 × 10^−17^ M^2^<AgBr=5.4 × 10^−13^ M^2^<AgCl=1.77 × 10^−10^ M^2^), i.e. a higher tendency to form solid AgX on the Ag surface, and a higher photosensitivity (AgI>AgBr>AgCl) in the counter-half-reaction for the recycling of the Ag surface atoms (Ag^+^→Ag).

We confirmed the role of photosensitive Ag salts for photorecycling of Ag atoms as electron donors in quantitative control experiments using silver halides (Supplementary Figs 13–15). According to the proposed reaction mechanism, Br^−^ and I^−^ should be even more active than Cl^−^ in this reaction due to their lower water solubility (*K*_SP_=5.4 × 10^−13^ and 8.52 × 10^−17^ M^2^ respectively) and the higher photosensitivity^26^ of their corresponding silver salts (AgBr and AgI). We found that with the same concentration of Br^−^ and Cl^−^, more 4-NTP molecules are reduced to 4-ATP in the Br^−^-containing solution (Fig. 4a). In particular I^−^ shows an extremely high activity, even at much lower concentrations than those used for Cl^−^ and Br^−^ (30 μM versus 100 mM). The relative contribution of the product 4-ATP to the SERS spectra calculated according to the characteristic Raman bands of 4-NTP and 4-ATP at 1,573 and 1,591 cm^−1^, respectively, at different X^−^ concentrations, are shown in Fig. 4b. The activity of X^−^ for the hot electron-induced redox chemistry follows the trend I^−^>Br^−^>Cl^−^, which directly correlates with the solubility and photosensitivity of their corresponding Ag salts. In negative control experiments we also used other anions such as SO_4_^2−^ and PO_4_^3−^ (Supplementary Fig. 16). The corresponding Ag salts have a higher water solubility and/or low photosensitivity compared with the Ag halides and cannot compensate the hot holes by photorecycling of Ag atoms and therefore exhibit no photocatalytic activity in this hot electron-induced reaction.

The proposed oxidation half-reaction in Fig. 3 (**6**→**7**) is the photodissociation of AgX (photography). This recovers Ag atoms which then induce reduction by hot electrons (**1**→**2**). Alternatively, AgX may be directly converted to Ag^+^ (hole)+X·+e^−^ (**6**→**3**) and no hot electrons are required for recycling. In this particular reaction (4-NTP to 4-ATP) it is not possible to disentangle both processes since AgX is required for photorecycling, while metallic Ag is necessary for SERS monitoring. The characterization of the Ag superstructures after reaction can be found in Supplementary Figs 17 and 18. Therefore, we tested if hot electrons from the photorecycling Ag surface can be used in other redox reactions which do not require SERS monitoring. Results in Supplementary Fig. 19 show the catalytic activity of Ag nanoparticles in the photoreduction of yellow [Fe(III)(CN)_6_]^3−^ to colourless [Fe(II)(CN)_6_]^4−^. Also here the halide anion efficacy trended with I^−^>Br^−^>Cl^−^. In contrast, AgX alone (without metallic Ag) cannot catalyse the same reaction in the control experiments (Supplementary Fig. 20), indicating the key role of the photorecycling metallic Ag/AgX surface in the reduction process. These results further support the proposed general reaction mechanism.

## Discussion

We report on the discovery of a counter-half-reaction promoted reduction chemistry on silver surfaces without the need of chemical reducing agents. Key elements for driving this reaction are hot electrons, protons and halide anions. Hot electrons are generated from silver and transferred to molecules adsorbed on the metal surface. Protons serve as the hydrogen source. Halide ions are required for photorecycling the electron-donating silver atoms (Ag) from hot holes (h^+^=Ag^+^) after photodissociation of the insoluble silver halides present on the Ag surface (Supplementary Discussion). A series of control experiments demonstrates that without this photorecycling counter-half-reaction the six-hot electron reduction cannot proceed. The discovery was enabled by the use of *in situ* SERS spectroscopy, which allowed us to directly identify the reaction product on-line and in a label-free approach. This unexpected photocatalytic route paves the way for exciting new opportunities for light-to-energy conversion schemes employing silver as a significantly cheaper catalyst compared with other noble metals such as Au, Pt and Pd.

## Methods

### Synthesis of monodisperse Ag nanoparticles

A modified method from ref. 27 was used to synthesize Ag nanoparticles. To synthesize ∼25 nm small Ag nanoparticles, 23 ml glycerol was mixed with 27 ml water in a 100 ml flask and heated up to 104 °C under vigorous magnetic stirring. Sodium citrate (10 mg) was dissolved in 1 ml water and added to solution in the flask. One millilitre of 0.5% AgNO_3_ aqueous solution was subsequently added. After 1 h, the reaction system was cooled down and yellow brown colloid was collected. The obtained ∼25 nm Ag nanoparticles were used as the seeds to produce ∼100 nm Ag nanoparticles. In a 250 ml flask, 580 mg of polyvinylpyrrolidone K30 was added to 138 ml water together with 23 ml glycerol. Seeds colloid (2.5 ml; 25 nm Ag nanoparticles) was added under stirring. After 20 s, 1.15 ml of a mixture of 20 mg AgNO_3_, 1 ml water and 220 μl ammonium hydroxide (25%) solution, was added to the flask together with 92 ml aqueous solution containing 36.8 mg ascorbic acid. The growth finished after 1 h. A milky white colloid was obtained. Fresh Ag nanoparticles are preferred for the synthesis of the plasmonic superstructures.

### Synthesis of Ag superstructures

The 100 nm Ag nanoparticles were used as cores of the superstructures. The surface of these large Ag nanoparticles was first coated with 3-mercaptopropyltrimethoxysilane (MPTMS) by adding 10 μl ethanolic MPTMS solution (10%) to 1 ml Ag colloid. This mixture was incubated in a temperature-controlled shaker (Eppendorf, Thermomixer comfort) at 50 °C for 1 h (750 r.p.m.). Then the nanoparticles were washed two times with water and resuspended in 1 ml water. An aqueous 0.054% sodium silicate solution (6 μl) was added to the colloid and the mixture was incubated at 90 °C for 1 h (750 r.p.m.). After centrifugation the ultrathin glass shell-isolated Ag cores were resuspended in 1 ml isopropanol. An aqueous ammonium hydroxide solution (50 μl, 25%) and ethanolic MPTMS solution (5 μl, 10%) were added together to the obtained glass shell-isolated Ag core suspension and then incubated at room temperature. After 30 min, the products were washed two times with isopropanol and resuspended in 1 ml isopropanol. The obtained suspension was mixed with 1 ml of 25 nm Ag satellite nanoparticles and then sonicated (ultrasonic bath Elmasonic P 60 H, 60% power) at room temperature for 20 min. Unbounded small satellite nanoparticles were removed after overnight incubation. For removal of the small satellite particles the mixture was centrifuged at relative centrifugal force (RCF) 600*g* for 10 min and the supernatant was discarded. The superstructures were resuspended in 1 ml water. After repeating the washing steps two times the final products were resuspended in 100 μl water.

### Hot electron-induced reduction from 4-NTP to 4-ATP

Ten microlitre of 10 mM ethanolic 4-NTP solution was added to 1 ml aqueous colloidal suspension of the Ag core/satellite superstructures with an ultrathin silica shell around the Ag core. The mixture was incubated overnight at room temperature to form a SAM of 4-NTP on the surface of the small satellites. Then the Ag superstructures were washed two times with water and resuspended in 1 ml water. To perform the hot electron-induced reaction with protons and halide anions, aqueous sulfuric acid and sodium or potassium halide, respectively, were added to 1 ml of the colloidal superstructures, with a final volume of 2 ml and proton concentration of 1 M. The 632.8 nm line from a He–Ne laser was employed for initiating the redox reaction and for the excitation of the SERS spectra. A WITec Alpha 300R microscope equipped with a monochromator (600 grooves per mm grating) and an EM-CCD (Andor) was used for the detection of the SERS spectra.

### Materials

Silver nitrite, sodium citrate, L-ascorbic acid, sodium silicate, MPTMS, tetraethylorthosilicate (TEOS), 4-ATP, sodium borohydride, ammonium hydroxide (25%), isopropanol, sulfuric acid, hydrochloric acid, phosphoric acid, sodium chloride, sodium bromide and potassium iodide were purchased from Sigma/Aldrich/Fluka. 4-NTP was purchased from Fluorochem. 4-MBA was purchased from TCI. Glycerol was purchased from AppliChem. Polyvinylpyrrolidone K30 was purchased from Carl Roth. All chemical reagents were used as received without further purification.

## Additional information

**How to cite this article:** Xie, W. & Schlücker, S. Hot electron-induced reduction of small molecules on photorecycling metal surfaces. *Nat. Commun.* 6:7570 doi: 10.1038/ncomms8570 (2015).

## Supplementary Material

Supplementary Information Supplementary Figures 1-20, Supplementary Notes 1-4, Supplementary Discussion and Supplementary References

## References

[b1] Hartland G. V. Optical studies of dynamics in noble metal nanostructures. Chem. Rev. 111, 3858–3887 (2011). 21434614 10.1021/cr1002547

[b2] Lal S. *et al.* Tailoring plasmonic substrates for surface enhanced spectroscopies. Chem. Soc. Rev. 37, 898–911 (2008). 18443675 10.1039/b705969h

[b3] Jain P. K., Huang X. H., El-Sayed I. H. & El-Sayed M. A. Noble metals on the nanoscale: optical and photothermal properties and some applications in imaging, sensing, biology, and medicine. Acc. Chem. Res. 41, 1578–1586 (2008). 18447366 10.1021/ar7002804

[b4] Linic S., Christopher P. & Ingram D. B. Plasmonic-metal nanostructures for efficient conversion of solar to chemical energy. Nat. Mater. 10, 911–921 (2011). 22109608 10.1038/nmat3151

[b5] Christopher P., Xin H. L. & Linic S. Visible-light-enhanced catalytic oxidation reactions on plasmonic silver nanostructures. Nat. Chem. 3, 467–472 (2011). 21602862 10.1038/nchem.1032

[b6] Lee J. *et al.* Plasmonic photoanodes for solar water splitting with visible light. Nano Lett. 12, 5014–5019 (2012). 22916955 10.1021/nl302796f

[b7] Manjavacas A., Liu J. G., Kulkarni V. & Nordlander P. Plasmon-induced hot carriers in metallic nanoparticles. ACS Nano 8, 7630–7638 (2014). 24960573 10.1021/nn502445f

[b8] Mukherjee S. *et al.* Hot electrons do the impossible: plasmon-induced dissociation of H_2_ on Au. Nano Lett. 13, 240–247 (2013). 23194158 10.1021/nl303940z

[b9] Ingram D. B. & Linic S. Water splitting on composite plasmonic-metal/semiconductor photoelectrodes: evidence for selective plasmon-induced formation of charge carriers near the semiconductor surface. J. Am. Chem. Soc. 133, 5202–5205 (2011). 21425795 10.1021/ja200086g

[b10] Seh Z. W. *et al.* Janus Au-TiO_2_ photocatalysts with strong localization of plasmonic near-fields for efficient visible-light hydrogen generation. Adv. Mater. 24, 2310–2314 (2012). 22467121 10.1002/adma.201104241

[b11] Giugni A. *et al.* Hot-electron nanoscopy using adiabatic compression of surface plasmons. Nat. Nanotechnol. 8, 845–852 (2013). 24141538 10.1038/nnano.2013.207

[b12] Haynes C. L., McFarland A. D. & Van Duyne R. P. Surface-enhanced Raman spectroscopy. Anal. Chem. 77, 338A (2005).

[b13] Lee H. M., Jin S. M., Kim H. M. & Suh Y. D. Single-molecule surface-enhanced Raman spectroscopy: a perspective on the current status. Phys. Chem. Chem. Phys. 15, 5276–5287 (2013). 23525118 10.1039/c3cp44463e

[b14] Nie S. M. & Emory S. R. Probing single molecules and single nanoparticles by surface-enhanced Raman scattering. Science 275, 1102–1106 (1997). 9027306 10.1126/science.275.5303.1102

[b15] Le Ru E. C. & Etchegoin P. G. in Principles of Surface-Enhanced Raman Spectroscopy Elsevier (2009).

[b16] Schlücker S. Surface-enhanced Raman spectroscopy: concepts and chemical applications. Angew. Chem. Int. Ed. 53, 4756–4795 (2014). 10.1002/anie.201205748 24711218

[b17] van Schrojenstein Lantman E. M. *et al.* Catalytic processes monitored at the nanoscale with tip-enhanced Raman spectroscopy. Nat. Nanotechnol. 7, 583–586 (2012). 22902959 10.1038/nnano.2012.131

[b18] Sun M. T. & Xu H. X. A novel application of plasmonics: plasmon-driven surface-catalyzed reactions. Small 8, 2777–2786 (2012). 22777813 10.1002/smll.201200572

[b19] Xie W., Herrmann C., Kömpe K., Haase M. & Schlücker S. Synthesis of bifunctional Au/Pt/Au core/shell nanoraspberries for in situ SERS monitoring of platinum-catalyzed reactions. J. Am. Chem. Soc. 133, 19302–19305 (2011). 22053855 10.1021/ja208298q

[b20] Huang J. F. *et al.* Site-specific growth of Au-Pd alloy horns on Au nanorods: a platform for highly sensitive monitoring of catalytic reactions by surface-enhanced Raman spectroscopy. J. Am. Chem. Soc. 135, 8552–8561 (2013). 23675958 10.1021/ja4004602

[b21] Xie W., Walkenfort B. & Schlücker S. Label-free SERS monitoring of chemical reactions catalyzed by small gold nanoparticles using 3D plasmonic superstructures. J. Am. Chem. Soc. 135, 1657–1660 (2013). 23186150 10.1021/ja309074a

[b22] Joseph V. *et al.* Characterizing the kinetics of nanoparticle-catalyzed reactions by surface-enhanced raman scattering. Angew. Chem. Int. Ed. 51, 7592–7596 (2012). 10.1002/anie.201203526 22806949

[b23] Zhang Q. F., Blom D. A. & Wang H. Nanoporosity-enhanced catalysis on subwavelength Au nanoparticles: a plasmon-enhanced spectroscopic study. Chem. Mater. 26, 5131–5142 (2014).

[b24] Li J. F. *et al.* Shell-isolated nanoparticle-enhanced Raman spectroscopy. Nature 464, 392–395 (2010). 20237566 10.1038/nature08907

[b25] Mohri N., Inoue M., Arai Y. & Yoshikawa K. Kinetic study on monolayer formation with 4-aminobenzenethiol on a gold surface. Langmuir 11, 1612–1616 (1995).

[b26] Greenwood N. N. & Earnshaw A. in Chemistry of the Elements Butterworth-Heinemann (1997).

[b27] Steinigeweg D. & Schlücker S. Monodispersity and size control in the synthesis of 20–100 nm quasi-spherical silver nanoparticles by citrate and ascorbic acid reduction in glycerol–water mixtures. Chem. Commun. 48, 8682–8684 (2012). 10.1039/c2cc33850e 22822486

