# Supplementary material for: Hot electron-induced reduction of small molecules on photorecycling metal surfaces
Source: Nat Commun. 2015 Jul 3;6:7570. doi: 10.1038/ncomms8570 (PMC4506517; doi:10.1038/ncomms8570)
Supplement: Supplementary Information — Supplementary Figures 1-20, Supplementary Notes 1-4, Supplementary Discussion and Supplementary References [file ncomms8570-s1.pdf]

Supplementary Figures

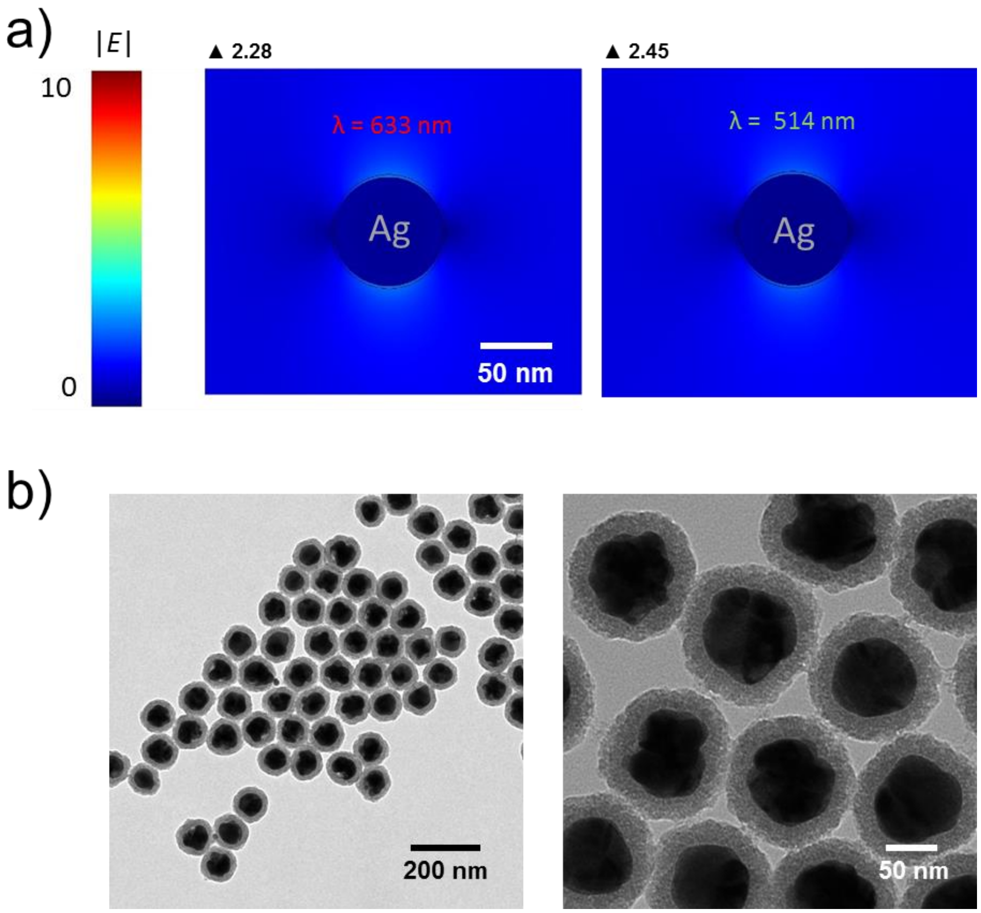

Supplementary figure 1. a, FEM simulation of |E| distributions in a 70 nm Ag nanoparticle upon excitation at 633 and 514 nm, respectively. The corresponding SERS enhancement factors $|E|^4$ are 27 and 36, respectively, which is negligible for SERS applications. **b,** TEM images of 20 nm silica-coated 70 nm Ag nanoparticles. The growth of the shell is performed very carefully to avoid aggregation so that only one nanoparticle monomer can be found in each shell. In this case the minimum distance between two adjacent Ag nanoparticles is 40 nm.

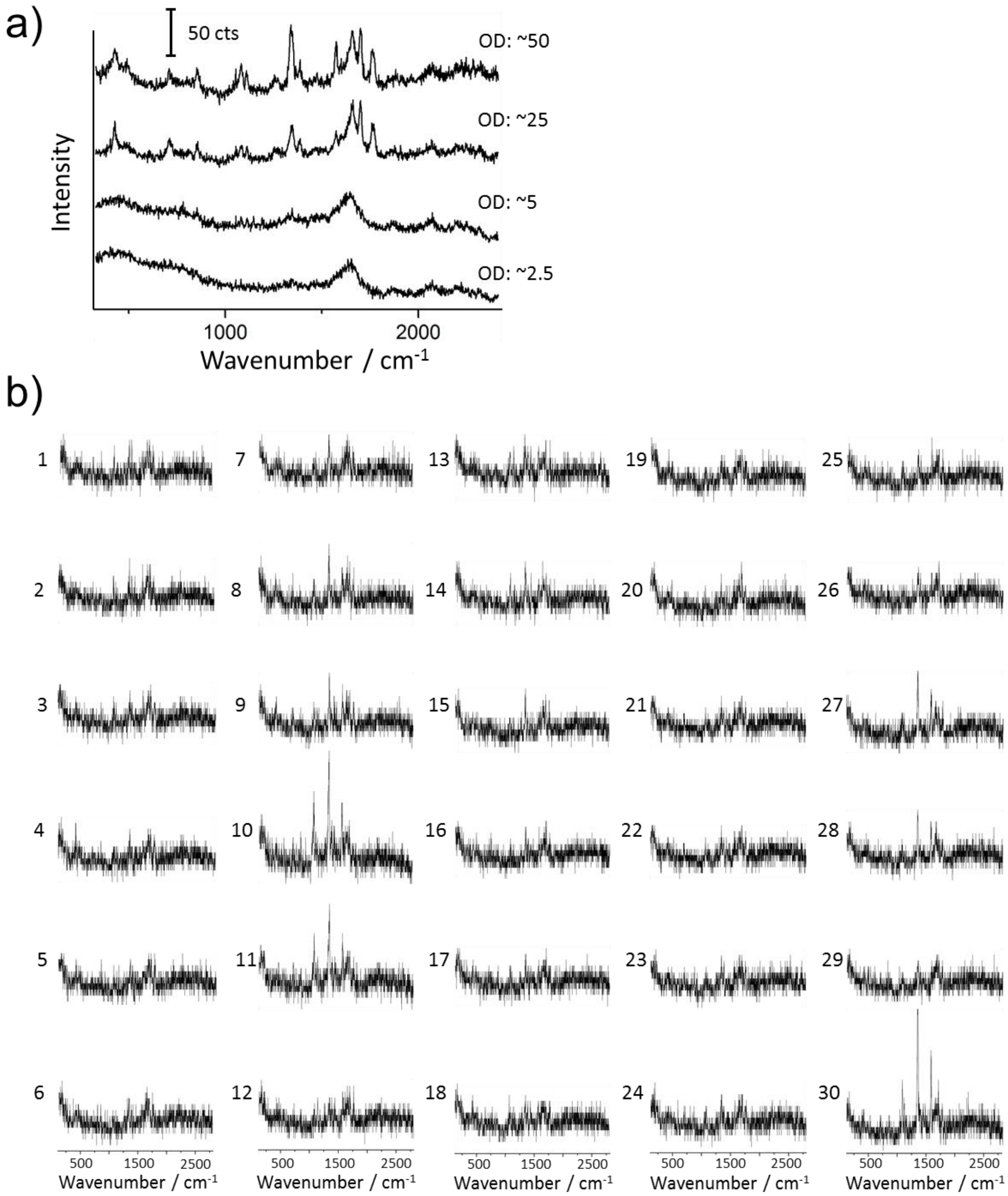

Supplementary figure 2. a, SERS spectra of 4-NTP from colloidal suspension of 20 nm glass shell encapsulated 70 nm Ag nanoparticles. The 4-NTP molecules are pre-coated on the Ag nanoparticle surface before the encapsulation with a silica shell. Due to the shell protection, no SERS signal was detected at OD 2.5 and 5. A very weak signal (~ 50 CCD counts for 30 s integration time) was detected at an

extremely high particle density (OD 50), equivalent to about 500 million particles per µl. **b,** SERS spectra obtained from the sample with OD value 50 at an integration time of 1 s. Only 7 out of 30 spectra exhibit a relatively strong SERS signal. There were about 500 million particles in the detection volume for all the spectra, indicating that even the weak signal from the highly concentrated monomer suspension is from very few clusters.

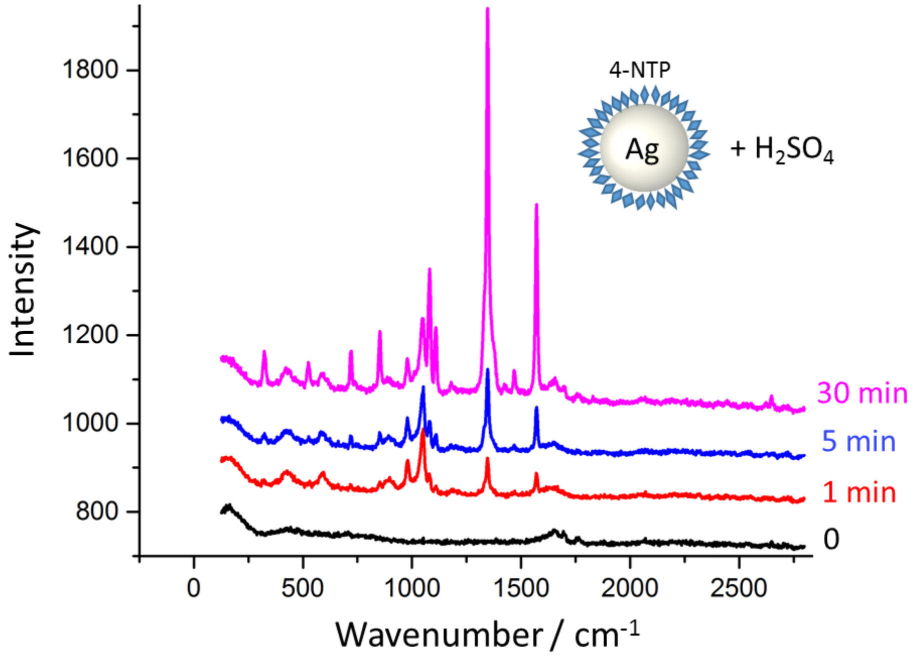

Supplementary figure 3. SERS spectra from 4-NTP SAM-coated 70 nm Ag nanoparticles (without glass shells) in 0.05 M H_2SO_4 solution. The signal was recorded at different times after the addition of the acid to the colloid (OD 2.5) to investigate the increasing SERS activity during nanoparticle aggregation.

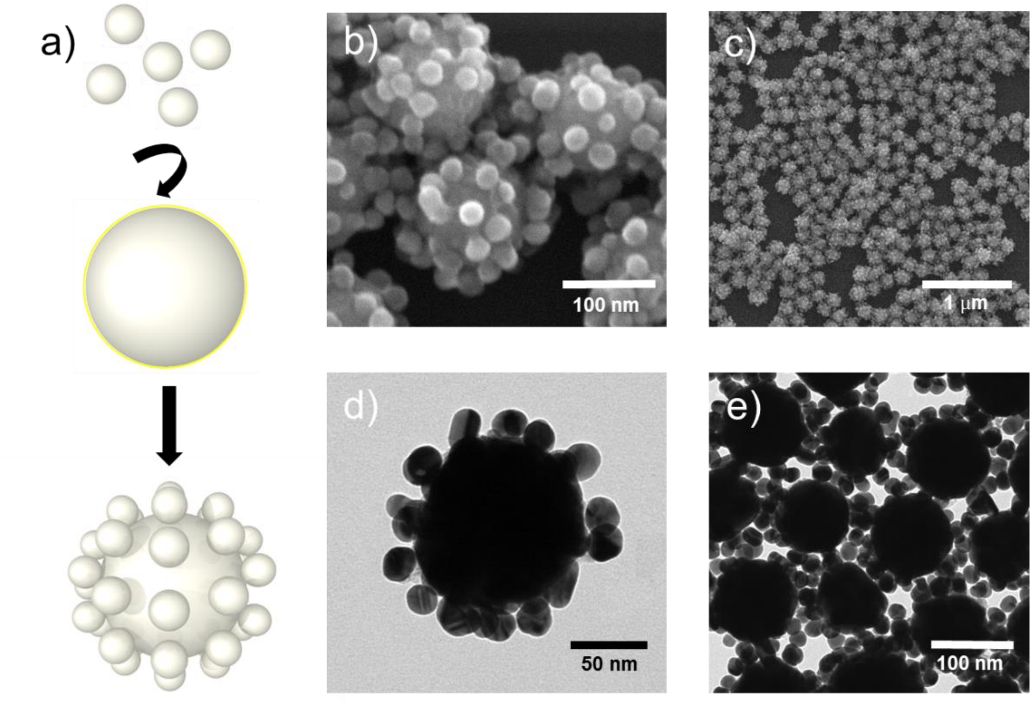

Supplementary figure 4. a, Schematic representation of superstructure synthesis. **b-c**, SEM images of the Ag superstructures. **d-e**, TEM images of the Ag superstructures. Both SEM and TEM images show monodisperse superstructures with high satellite surface coverage.

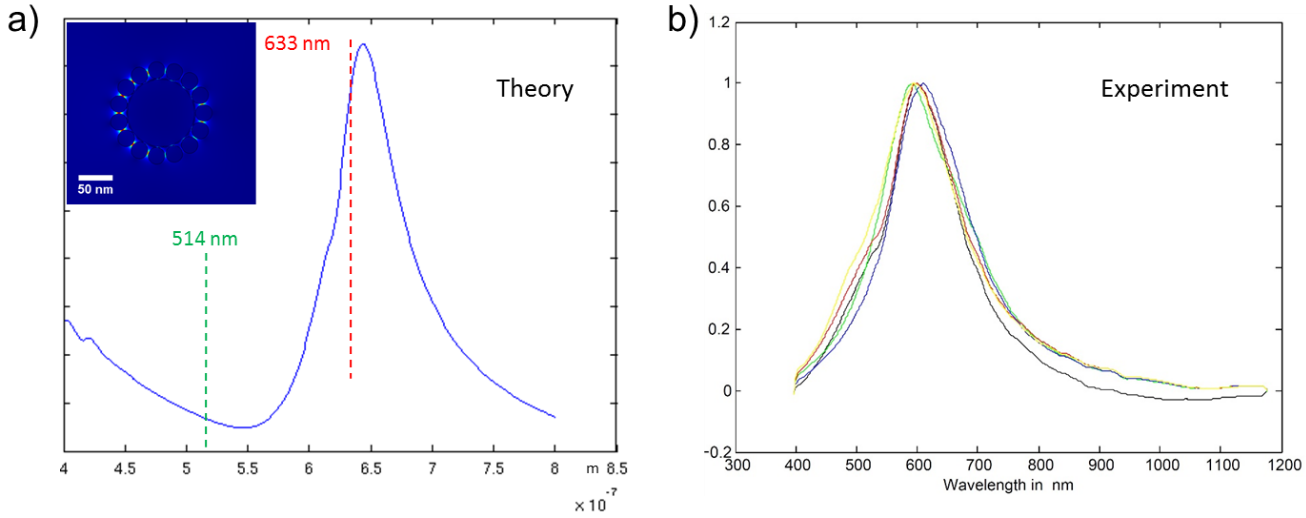

Supplementary figure 5. a, Finite element method (FEM) simulation of the scattering spectrum from a single Ag superstructure in visible range. The maximum intensity is close to the red laser excitation at 633 nm. **b**, Single Ag superstructure scattering spectra measured from 5 individual Ag superstructures. Both shape and peak position correlate reasonably well with the simulation.

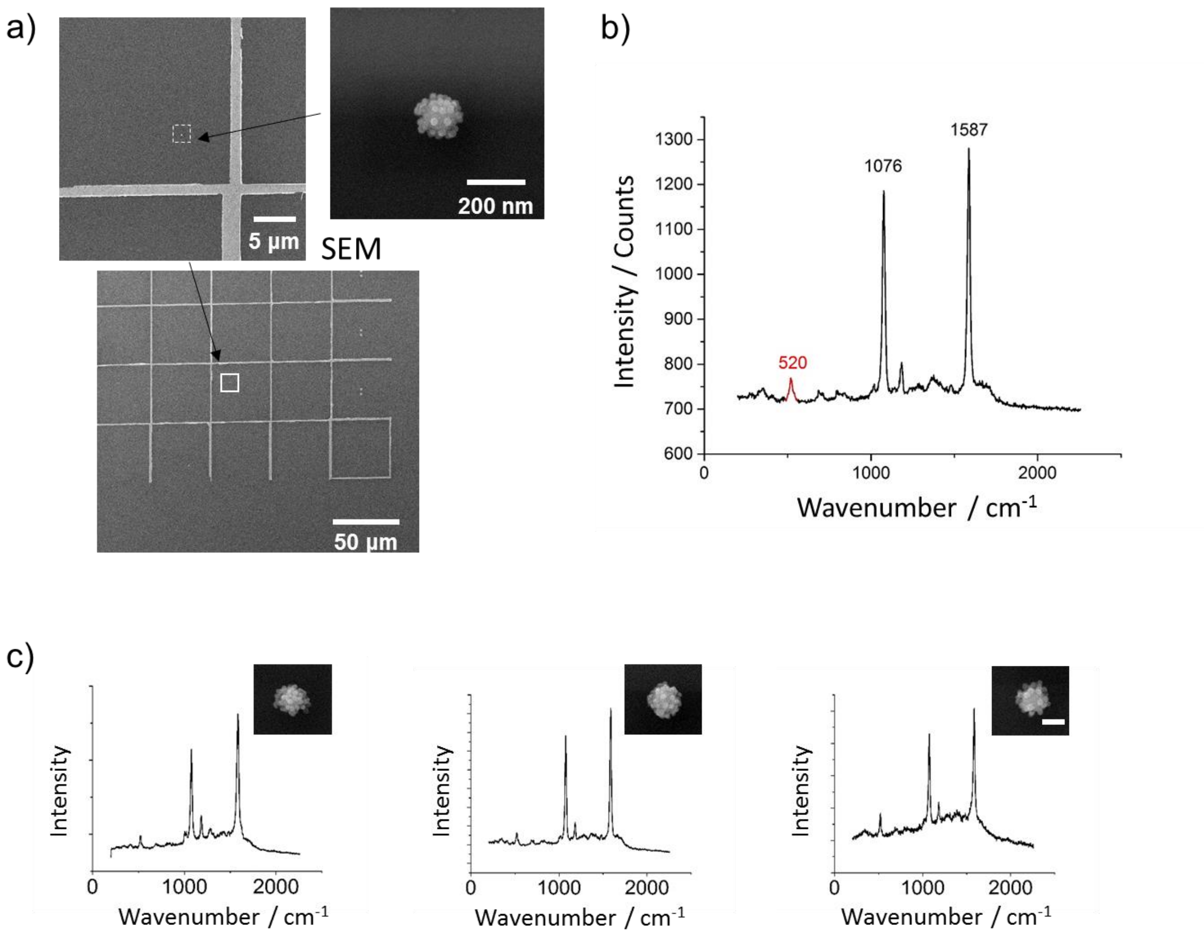

Supplementary figure 6. a, SEM images of a single Ag superstructure on a framed Si wafer for single particle SERS experiments. The single particle can be found again under the optical microscope for Raman measurements. **b**, SERS spectrum of 4-mercaptobenzoic acid (4-MBA) on the single Ag superstructure shown in the SEM images. The Si peak occurs at ca. 520 cm^{-1}. The laser power is about 0.6 mW (wavelength: 632.8 nm) and the integration time is 36.5 ms. **c**, Several single particle SERS spectra obtained from other individual Ag superstructures on the Si wafer and corresponding SEM images (insert). The scale bar is 100 nm.

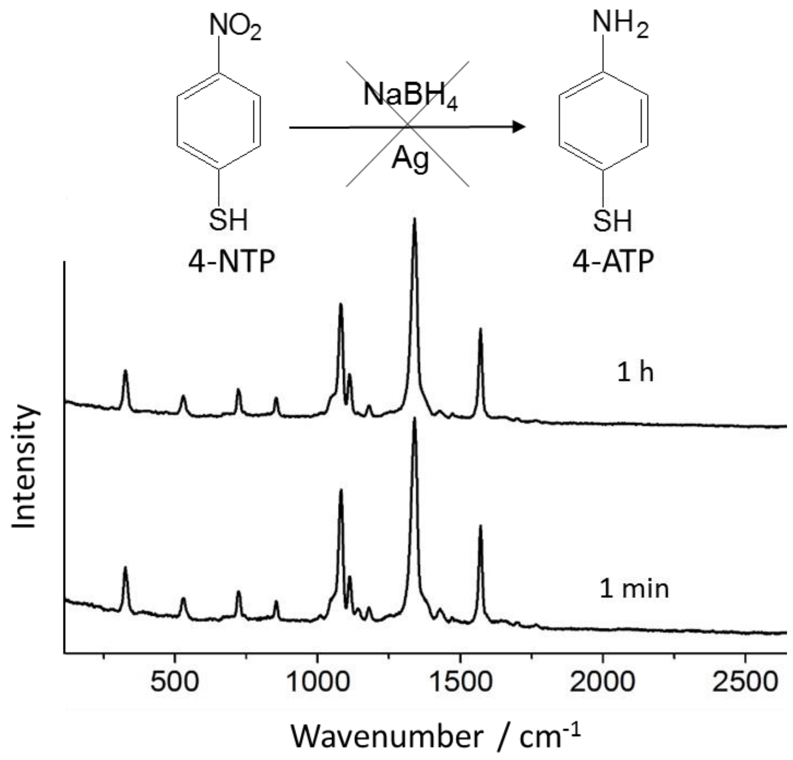

Supplementary figure 7. SERS spectra from 4-NTP SAM-coated Ag superstructures after addition of sodium borohydride (1 mM final concentration) to the colloidal suspension. No 4-ATP signal has been detected 1 hour after the addition, indicating that Ag cannot catalyze the hydride reaction from 4-NTP to 4-ATP.

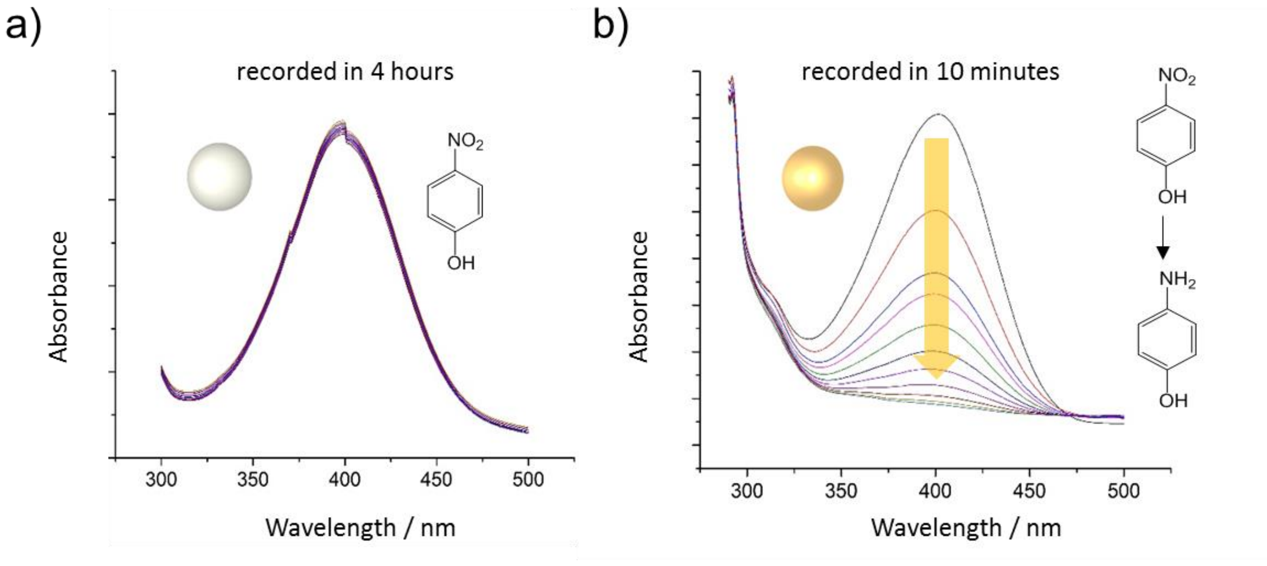

Supplementary figure 8. a, UV-Vis absorption spectra of 4-nitrophenol mixed with aqueous sodium borohydride solution together with Ag nanoparticles (the satellites in the plasmonic Ag superstructures) recorded in 4 hours. No catalytic reaction occurs. **b,** Positive control experiment using Au nanoparticles as the catalysts. The spectra were recorded in only 10 minutes and nearly all the 4-nitrophenol molecules were reduced.

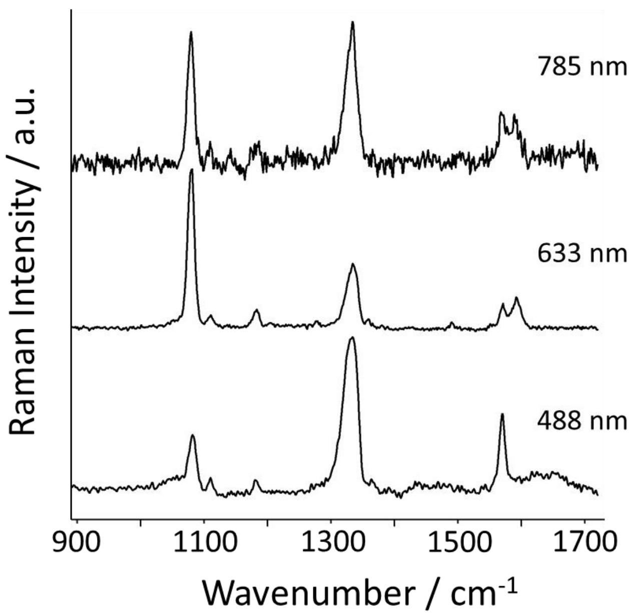

Supplementary figure 9. SERS spectra of the hot-electron reduction using different laser excitation wavelengths. Ag superstructures coated with a 4-NTP SAM were suspended in 0.2 M HCl solution for all the experiments. The spectrum obtained with 488 nm excitation was recorded on a Bruker Senterra Raman microscope and a WITec Alpha 300R microscope was used for 785 nm SERS measurement. Although the experimental conditions are different due to the wavelength dependent-SERS activity of the nanostructures and also the limitation from the setups/software, the 633 nm shows the highest reduction activity with shortest illumination time (10 s). In contrast, 488 nm excitation leads to no activity even with the longest illumination time (120 s).

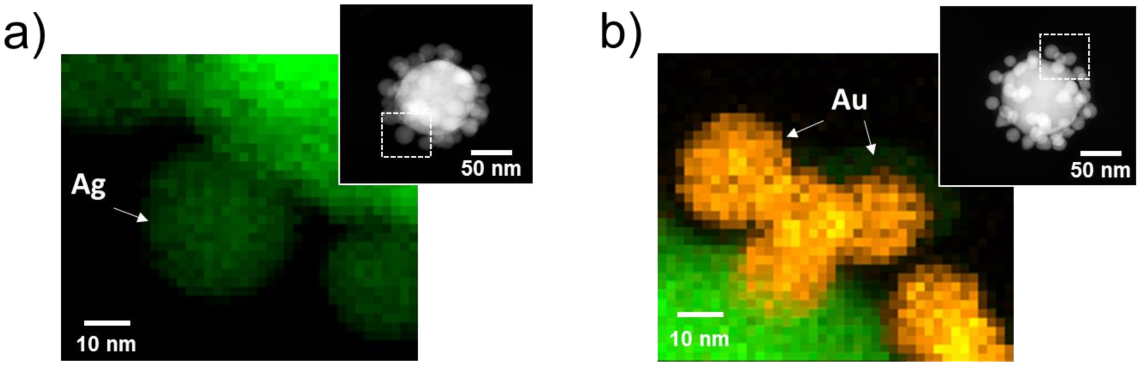

Supplementary figure 10. EDS element maps for Au and Ag in the monometallic Ag (**a**) and bimetallic Au@Ag (**b**) superstructures together with the corresponding STEM images (inset).

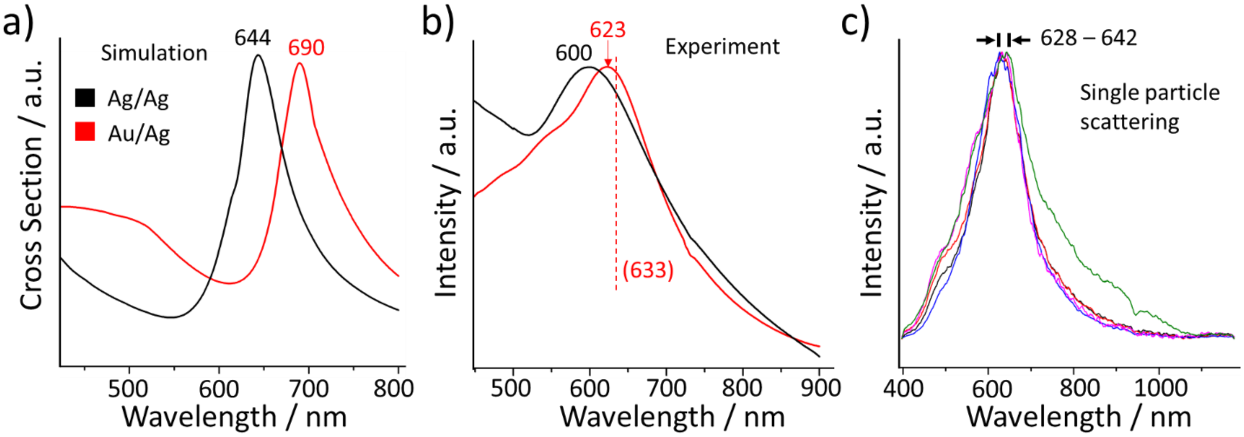

Supplementary figure 11. a) Finite element method (FEM) simulation of the extinction spectra of Ag core Ag satellite (black) and Ag core Au satellite (red) superstructures. b) Extinction spectra of the superstructures with Ag (black) and Au (red) satellites in colloidal suspension. c) Single-particle scattering spectra from 5 individual Ag core/Au satellite superstructures.

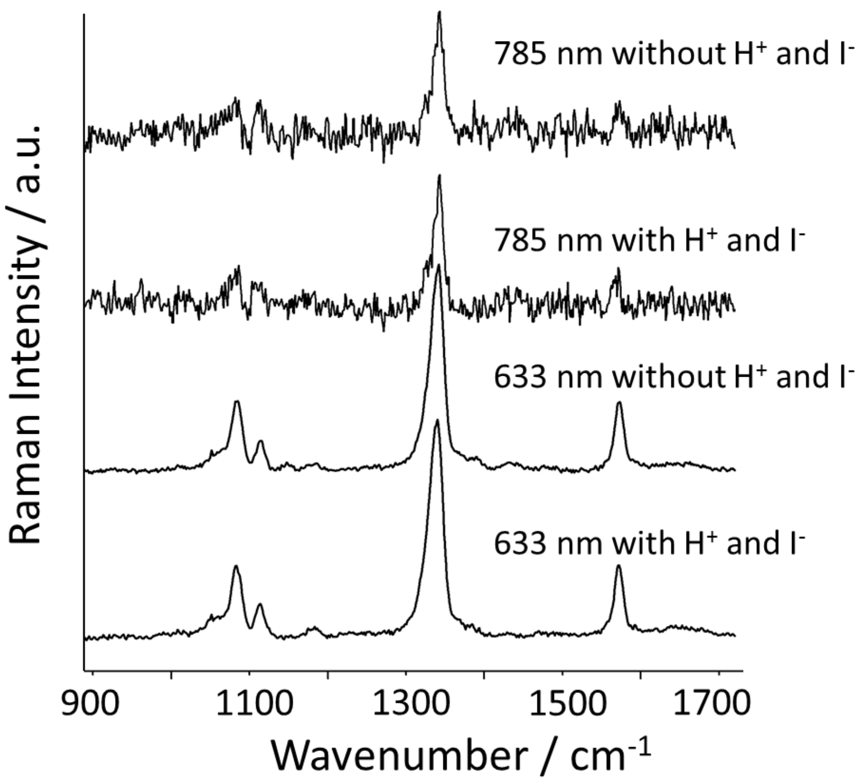

Supplementary figure 12. SERS spectra of 4-NTP coated Ag core Au satellite superstructures with and without the presence of protons and I^- (instead of HCl) using 633 and 785 nm laser as the excitation beam, respectively. The spectra were recorded at the same condition (laser power: ~18.6 mW and accumulation time: 10 s). The low signal-to-noise ratio of the spectra can be attributed to the weak plasmonic activity at 785 nm excitation (see Supplementary Figure 11).

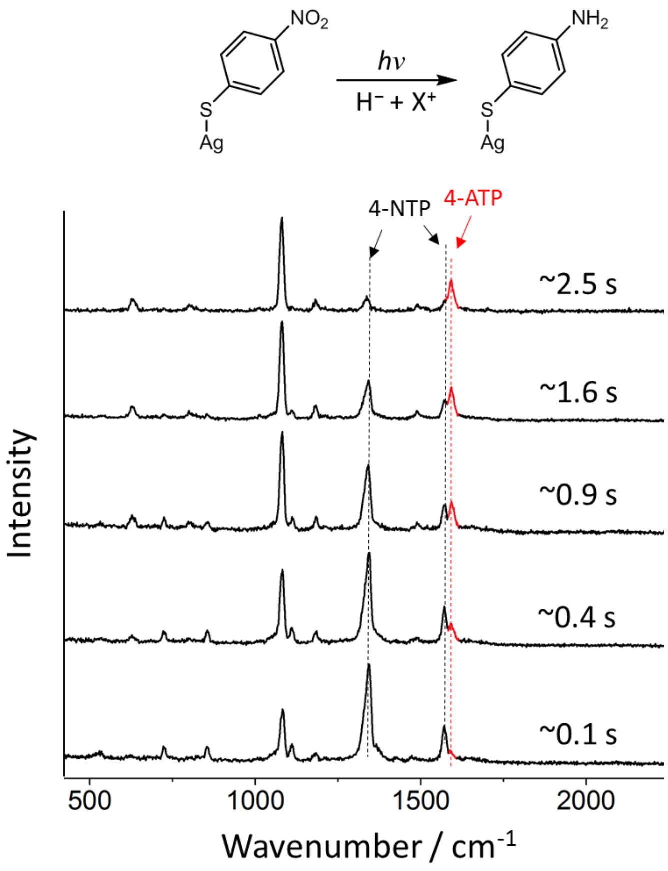

Supplementary figure 13. Quantitative SERS monitoring of the hot electron-induced reduction from 4-NTP to 4-ATP. The colloidal sample containing 1 M HCl was injected into a microfluidic channel for SERS experiments. All adjustment and calibration steps were performed at a fast sample flowing speed. The quantitative SERS signal was recorded at different laser exposure times when the sample flow was stopped. These results show that the reaction is initiated by the laser illumination.

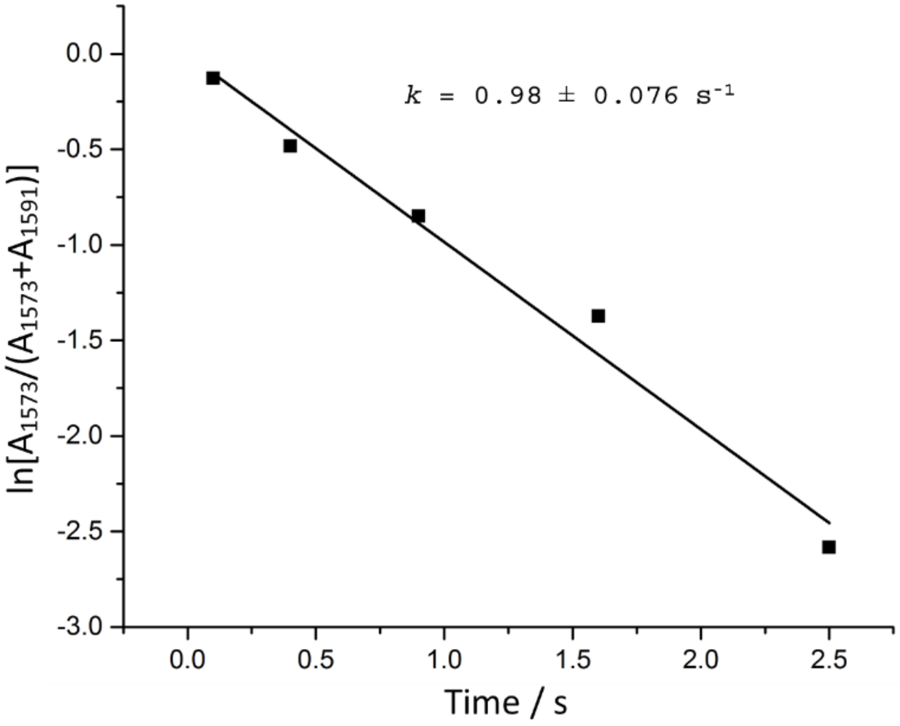

Supplementary figure 14. Calculation of the rate constant for the photo-catalytic reduction of 4-NTP with hot electrons generated on a photo-recycling Ag surface based on the band of 4-NTP at 1573 cm^{-1} and of 4-ATP at 1591 cm^{-1} in the SERS spectra. The original SERS spectra are shown in Supplementary Figure 13. Cl^{-} was used as the electron donor in this experiment.

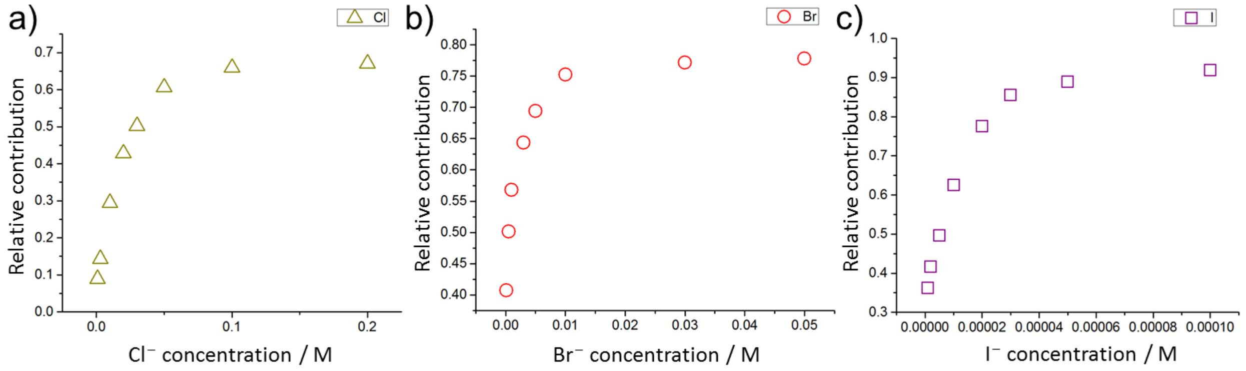

Supplementary figure 15. Relative contribution of the product (4-ATP) to the SERS spectra from the reaction suspension as a function of chloride (**a**), bromide (**b**) and iodide (**c**) anion concentrations. All the measurements were performed in 0.5 M aqueous H_2SO_4 solutions. These results are summarized in Figure 4b in the main text.

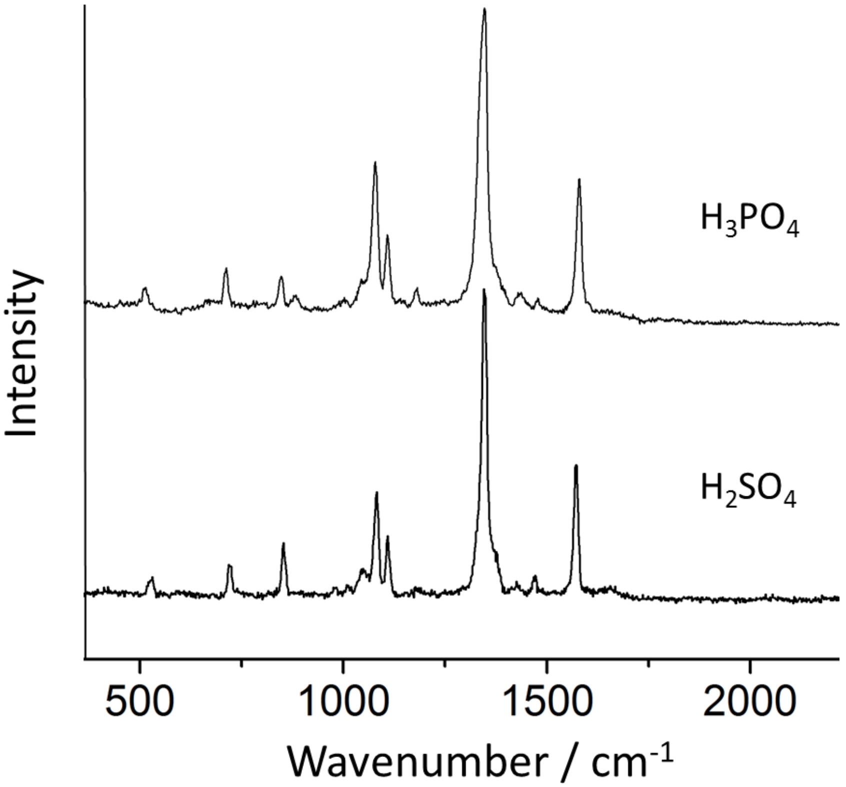

Supplementary figure 16. SERS spectra from 4-NTP molecules on Ag superstructures in aqueous H_3PO_4 (top) and H_2SO_4 (bottom) solution (pH = 0). No 4-ATP signal was detected in both cases.

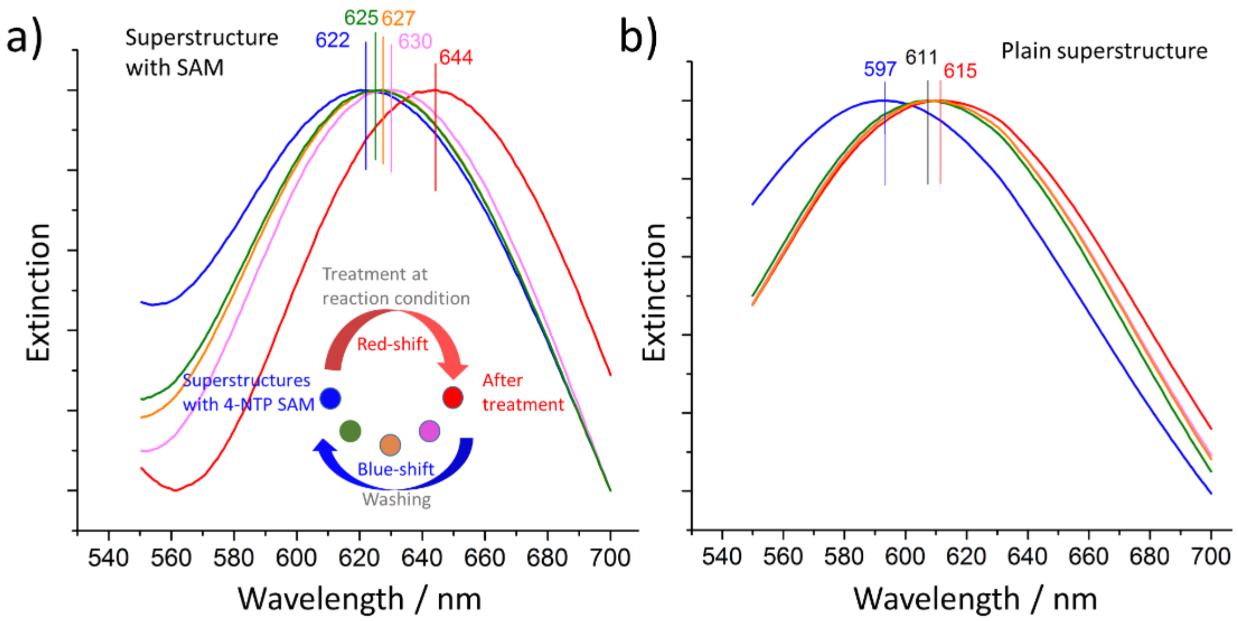

Supplementary figure 17. a) Extinction spectra of Ag superstructures covered with a 4-NTP SAM before (blue curve) and after (red curve) treatment with reaction solution (3×10^{-5} M KI, 0.5 M H_2SO_4 and illumination under 633 nm laser for 10 minutes). The treatment results in a 22 nm red-shift of the extinction band. However, after washing (centrifugation and resuspending the particles) the colloid with water, the extinction band shifted back (blue shift). Pink, orange and blue graphs correspond to the samples after 1, 2 and 3 washing steps, respectively. b) Control experiment using Ag superstructures without a molecular SAM. The extinction band red-shifted ~14 nm after the same treatment and washing process.

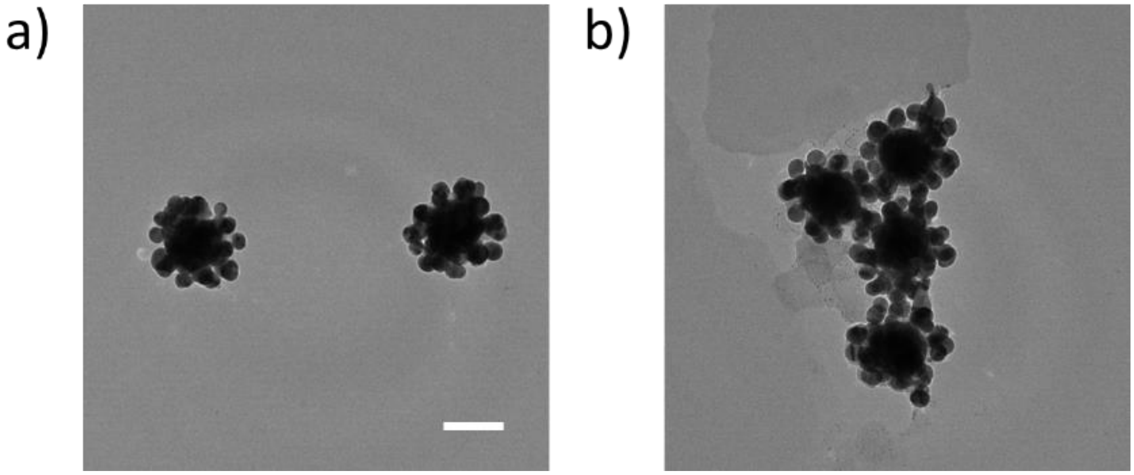

Supplementary figure 18. TEM images of the Ag superstructures before (a) and after (b) reaction (scale bar 100 nm). The samples were prepared by centrifugation of the particles from the aqueous suspension and then resuspended in ethanol. The morphology of the superstructure does not change after the reaction. The dirt in image b is probably from the reaction solution (acid and salt).

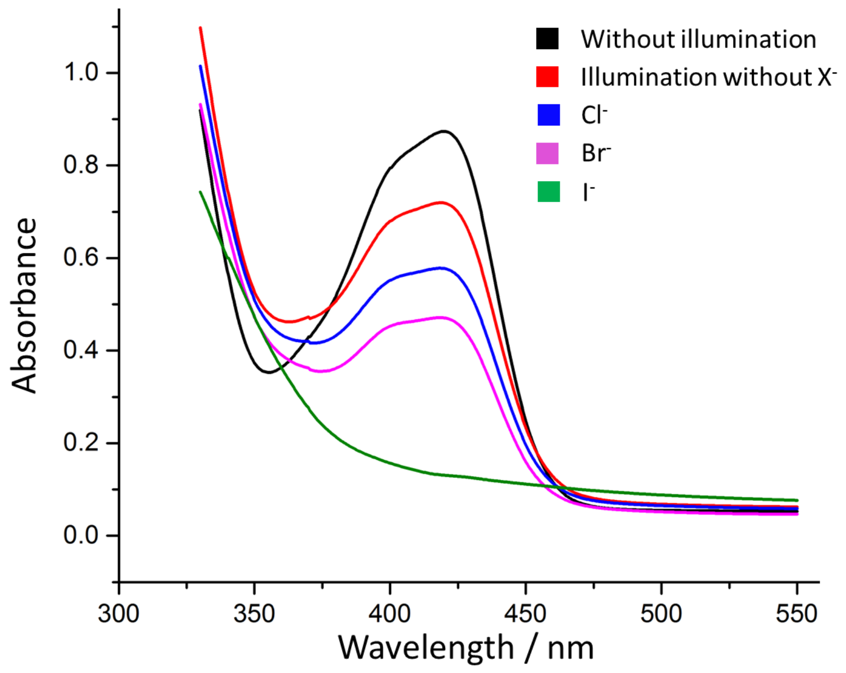

Supplementary figure 19. UV-Vis absorption spectra of the photo-catalytic reduction from yellow $[Fe(III)(CN)_6]^{3-}$ to colorless $[Fe(II)(CN)_6]^{4-}$. The absorbance of $[Fe(III)(CN)_6]^{3-}$ at ~420 nm decrease after the reduction. The reaction mixture containing 8.4×10^{-4} M $[Fe(III)(CN)_6]^{3-}$, 2.2×10^{-2} M halide anions and about 2.2×10^{10} particles/ml Ag NPs was illuminated under a mercury lamp for 20 minutes (power density 400 W/m^2) before the measurement. The absorbance does not change without light illumination (black curve, in the presence of halide and Ag).

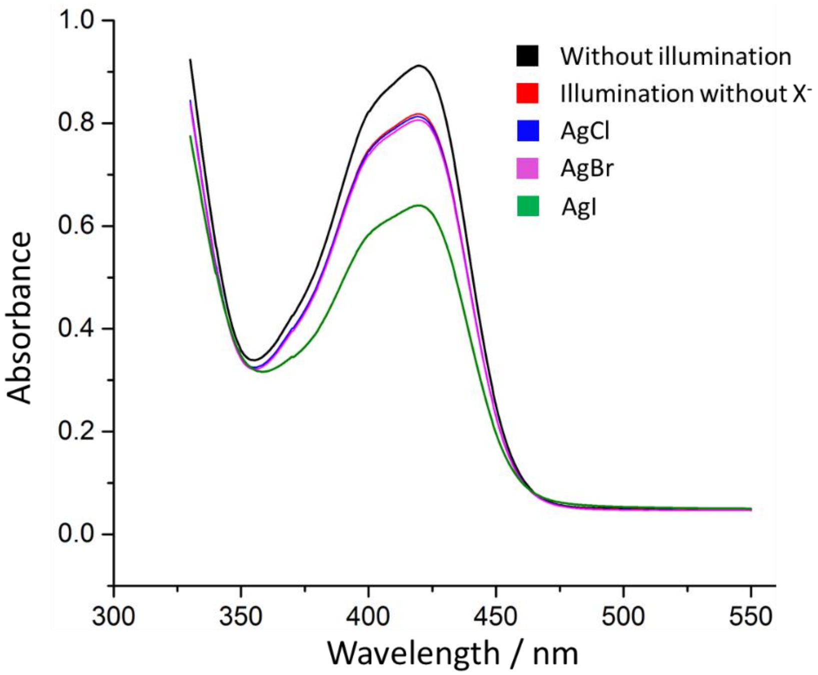

Supplementary figure 20. UV-Vis absorption spectra of the photo-catalytic reduction from $[Fe(III)(CN)_6]^{3-}$ to $[Fe(II)(CN)_6]^{4-}$ using AgX as the catalysts. The reaction condition is the same like in the experiments with Ag colloids (Supplementary Figure 9) except the using of $AgNO_3$ (2.2×10^{-5} g/ml) instead of the metallic Ag (the concentration of Ag element is the same). In this case AgX is formed in the reaction mixture. AgCl and AgBr show no activity for this reaction. The low activity of AgI can be attributed to the photo-reaction of AgI with $[Fe(III)(CN)_6]^{3-}$.

Supplementary Note 1

Plasmonic activity of Ag nanoparticle monomers. Silver is the most plasmonically active metal. It is therefore very promising to use Ag to produce hot electrons for plasmon-induced chemical reactions. In our experiments, surface-enhanced Raman scattering (SERS) is the analytical method for monitoring the reactions on metal surface. Is it possible that the Ag nanoparticle monomers can already provide the required plasmonic activity for both hot electron generation and sufficient SERS enhancement? A theoretical study on individual Au nanoparticles has already demonstrated that spherical monomeric Au nanoparticle exhibits very low plasmonic activity[1], which is not sufficient for the required SERS measurement in practice. It is well known that hot spots, including nanogaps between particles and sharp tips on nanostars or AFM tips, are required for producing very intense SERS signals. In this context a number of metal nanostructures with high plasmonic activity, such as dimers, nanoshells, nanocages, and superstructures, have been synthesized for SERS experiments in various applications. However, it is still common to use spherical/subspherical noble metal nanoparticles as substrates to enhance the Raman signal, without considering the aggregation status of the nanoparticles during the collection of the SERS signal. However, many experimentalists employ nanoparticle monomers in their studies and observe sufficient SERS. One therefore may have the wrong impression that nanoparticle monomers alone can generate sufficient SERS signals. Here, the plasmonic activity of Ag nanoparticle monomers was investigated both theoretically and experimentally.

We simulated the incident electric field amplitude |E| upon plasmon excitation of 70 nm Ag nanoparticles using the finite element method (FEM)

(Supplementary Figure 1 a). The maximum |E| value at 514 and 633 nm excitation is only 2.45 and 2.28, corresponding to SERS enhancement factors of 36 and 27 ($|E|^4$), respectively, which are negligible enhancements in applications. Experimentally, the 60 nm Ag nanoparticles[2] were first coated with a SAM of 4-NTP, and then encapsulated with a very thick glass shell to avoid plasmonic coupling between individual nanoparticles. To ensure that the obtained nanoparticles are monomers, the experiments were performed in a very careful way, especially the washing steps. For example in each washing step the colloid was centrifuged at a very slow speed (RCF 600 g) only once to ensure minimum aggregation. The TEM images of the obtained 20 nm silica shell-encapsulated[3] Ag nanoparticles are shown in Supplementary Figure 1 b. We could not find even one particle with a dimer or other clusters encapsulated in the same shell. In this case the minimum distance between two particles is about 40 nm.

The SERS activity of the monomers was measured at different nanoparticle concentrations (Supplementary Figure 2 a). At optical density (OD) 2.5 and 5, which are actually already high concentration for SERS detection, no SERS signal is obtained (30 seconds integration time). A weak signal is detected at a very high concentration (OD 50). A series of SERS spectra was recorded with 1 sec integration time (Supplementary Figure 2 b). Seven out of 30 spectra have relatively strong SERS, while the remaining ones exhibit only a very weak signal. We attributed this to the diffusion of the very few clusters into and out of the detection focal volume of the laser. At this concentration (OD 50) there are about 500 million nanoparticles in the focal volume (~ 1 µl). It reasonable to assume that there are at least few clusters among these 500 million nanoparticles, although we cannot find them in TEM images with only thousands of particles on one

sample grid, which may contribute most of the SERS signal during the measurement. In other words, 60 nm Ag nanoparticle monomers do not produce a sufficient SERS signal in our experiments

Another positive control experiment was performed to show how SERS can be produced using Ag nanoparticles without glass protection. 70 nm silver nanoparticles were first coated with 4-NTP SAM and then suspended in 0.05 M H_2SO_4 solution. SERS signals were recorded within 30 minutes and a gradual increase of enhancement was observed (Supplementary Figure 3) due to aggregation of the nanoparticles. A conclusion can be made that monomers of Ag nanoparticles cannot provide the required SERS enhancement for monitoring of catalysis. For quantitative SERS monitoring and high signal reproducibility, nanostructures with high plasmonic activity are recommended as the SERS substrate (Supplementary Figure 4-6). Additionally, the high plasmonic activity is also a requirement for producing hot electrons in the redox process.

Supplementary Note 2

Catalytic activity of Ag in hydride reduction of 4-NTP. When we use noble metals as plasmonic substrates for optical or spectroscopic studies, in particular in single particle experiments, the prize of the metal is typically not relevant. However, in catalysis and in particular with respect to potential industrial applications, it is very important that a cheaper metal can be used to produce the same product or reduce/oxidize the same educt. Silver is much cheaper than Au and Pt: the price of Ag is roughly 1-2 % of the Au or Pt price. Therefore it is advantageous to use Ag instead of Au and Pt for heterogeneous catalysis. In the model reaction from 4-

NTP to 4-ATP reduced by sodium borohydride, Au and Pt have already showed their catalytic activity in several previous works[4-6]. To test if Ag can also catalyze the same reaction, we first incubated our Ag superstructures with 4-NTP molecules and coated the Ag surface with a SAM of the educt. Then sodium borohydride solution was added to the superstructure suspension as the reducing agent. However, no 4-ATP signal was observed in the SERS spectrum from the reaction (Supplementary Figure 7), indicating that Ag cannot catalyze this hydride reduction. We also used the model reaction from 4-nitrophenol to 4-aminophenol, which cannot be monitored by SERS due to the absence of the thiol surface-seeking groups. Again no reduction was detected in the UV-Vis absorption spectra after 4 hour-incubation of the reactants and catalysts (Supplementary Figure 8 a). In a positive control experiment this reaction was easily initiated when 10 nm Au nanoparticles were added (Supplementary Figure 8 b). Therefore we conclude that Ag has no catalytic activity in the classic hydride reduction from 4-NTP to 4-ATP. It has to be mentioned here that the Ag nanoparticles used in these negative control experiments should be synthesized and treated in a careful way, for example using high quality chemicals and solvents for the synthesis and washing steps, to avoid trace amount of catalytically active materials that may lead to false positive results.

Supplementary Note 3

Wavelength-dependent reduction. We have performed the experiments at 488 and 785 nm laser excitation. Ag superstructures with a 4-NTP SAM were suspended in aqueous solution of 0.2 M HCl before the SERS measurement. The measurements were performed using different microscopes and spectrometers.

The excitation power of the different lasers is comparable (16.8, 18.7 and 19.4 mW for 488, 785 and 633 nm laser, respectively). As shown in Supplementary Figure 9 below, 633 nm excitation shows the best activity for this photo-catalytic reduction (illumination time = signal accumulation time = 10 s). In contrast, 785 nm has a lower activity with 60 s minute illumination time. Surprisingly, no 4-ATP product can be detected even with 120 s illumination using the 488 nm laser line, although the superstructures are still SERS-active, indicating that the very high plasmonic activity of the Ag superstructures in the red region is required for this six-electron reduction. The different quality of the spectra can be attributed to both the different setups and the wavelength-dependent plasmonic activity of the nanostructure.

Supplementary Note 4

Control experiments using Au satellites. We synthesized hybrid Ag core-Au satellite superstructures (Supplementary Figure 10) for control experiments since the Au satellites might donate their hot electrons to the SAM on their surface. We first compared the extinction properties of both superstructures with Au and Ag satellites. We have performed both computer simulations and experiments on the plasmonic activity of the superstructures (Supplementary Figure 11). The simulations show that the Au/Ag superstructures have an extinction maximum at 690 nm (Supplementary Figure 11 a). Experimentally, the extinction maximum of Au/Ag satellite-core superstructure at the ensemble level in colloidal suspension is at 623 nm compared to 600 nm for the Ag/Ag superstructure (Supplementary Figure 11 b). The difference between theory and experiment is attributed to the

2D model used in the simulation. We also measured the scattering spectra of single superstructures (Supplementary Figure 11 c). The maximum intensity is between 628 and 642 nm. These results indicate that the LSPR property between Au/Ag and Ag/Ag is not very different and the 632.8 nm laser line is suitable for the excitation of both superstructures.

Furthermore, we have tested 785 nm laser excitation for the SERS control experiment using Au/Ag superstructures. The result in Supplementary Figure 12 shows that the Au satellites in the Au/Ag superstructures are not active at both 633 and 785 nm excitation with I^- as the electron donor (presence of the symmetric nitro stretching peak of 4-NTP at ~1340 cm^{-1}), which indicates that Au, in contrast to Ag, cannot catalyze this six-electron reduction from 4-NTP to 4-ATP.

Supplementary Discussion

In redox chemistry, half-reaction and the corresponding counter-half-reaction are mutually dependent. The hot electrons generated by the non-radiative decay of the plasmons on the metal surface can be used in reduction chemistry. However, it is limited by the high rate of charge-carrier recombination. For example, 4-NTP molecules can be reduced to 4,4'-dimercaptoazobenzene (DMAB)[7-9] on the Ag superstructure due to the hot electrons. In this case each 4-NTP molecule needs 4 hot electrons:

$$-NO_2 + 4e^- + 2H_2O \rightarrow (1/2)-N{=}N- + 4OH^- \quad (1)$$

Further reduction to 4-ATP (− NH_2) is not possible without additional electrons from outside. In other words, the plasmonic Ag surface acts as a water cannon on a fire truck, the hot electrons act as the water and the resonant light is the pump. Of course a water source such as a hydrant is needed when a large amount of water is required for the fire. In hot electron reduction chemistry, the oxidation half-reaction is the hydrant.

We found by accident that when Cl^- was added to the 4-NTP coated Ag superstructure suspension, the nitro groups can be reduced to amino groups (4-ATP) in a photocatalytic reaction (Supplementary Figure 13). We have calculated the reaction rate of the photo catalytic reduction and compared it with the previous works in which chemical reducing agents were used. As shown in Supplementary Figure 14, the photo-catalytic reduction of 4-NTP to 4-ATP on Ag follows a first order kinetics and the rate constant calculated according to the experimental result in Supplementary Figure 13 is (0.98 ± 0.076) s^{-1}, which is much larger than the value from the previous studies using chemical hydride reducing agents: 1.13×10^{-4} s^{-1} in Au-catalyzed hydride reduction[6], 1.1×10^{-2} s^{-1} in Pt-catalyzed hydride reduction[5] and 2.17×10^{-3} s^{-1} in Pd-catalyzed hydrogen reduction[10]. Therefore, the hot-electron-induced reaction using Ag superstructures is faster than the chemical reagent-induced reduction with other metals (Au, Pt and Pd).

It has been proposed that the formation and subsequently decomposition of the AgCl on the superstructure surface is the oxidation half-reaction which is responsible for the hot electron promotion to initiate the reduction of 4-NTP to 4-ATP. To support the mechanism, we have performed experiments using different

anions including, Br^-, I^-, PO_4^{3-}, and SO_4^{2-}. Due to high photo-sensitivity and very low water solubility of the silver halides ($K_{SP(AgBr)} = 5.4 \times 10^{-13}$ and $K_{SP(AgI)} = 8.52 \times 10^{-17}$), both Br^- and I^- show high activity in this reaction (Supplementary Figure 15). Supplementary Figure 16 shows the SERS spectra of 4-NTP on Ag superstructures in H_3PO_4 and H_2SO_4 aqueous solution. Because of the relatively higher solubility and much lower photo-sensitivity of Ag_3PO_4 and Ag_2SO_4 in comparison to the silver halides, during the excitation of hot electron-hole pairs, first Ag salts formation on the Ag surface is difficult and then the required decomposition is also not possible. Therefore PO_4^{3-} and SO_4^{2-} cannot act as the "hydrant" to establish the oxidation half-reaction on the Ag surface. We assume that other anions including NO_3^-, F^-, and CO_3^{2-} are not active for this reaction but we did not test them because they are unstable in the protic environment; H_2CO_3 will decompose into CO_2, HNO_3 will oxidize the Ag surface and HF can destroy the glass fluidic channel or cuvette.

We have characterized the superstructures after the reaction by UV-Vis extinction spectroscopy and TEM. The extinction band red-shifted for more than 20 nm after the reaction (Supplementary Figure 17). However, the band shifted back after washing several times with water (centrifugation and resuspending the particles), indicating that no obvious change in the nanostructure morphology occurs. A TEM image of the sample after the reaction is shown in Supplementary Figure 18 b. No evidence of any structural change was found in these superstructures. The small Ag NPs act as catalysts in the hot-electron induced photo-catalytic reduction, and therefore the reaction does not cause any damage on the superstructures.

Furthermore, a different reduction reaction which does not involve protons was used to confirm the proposed mechanism. We mixed $K_3[Fe(III)(CN)_6]$ with Ag NPs and halide anions in aqueous solution. After light illumination the yellow $[Fe(III)(CN)_6]^{3-}$ ions were reduced to colorless $[Fe(II)(CN)_6]^{4-}$ ions by the hot electrons produced on the Ag surface (Supplementary Figure 19). The reaction can be detected by UV-Vis absorption spectroscopy. Since SERS is not needed, we used standard Ag NPs instead of our complex Ag superstructures to further demonstrate the general applicability of the mechanism. This one-electron reduction could occur (red curve in Supplementary Figure 19) in the absence of halide anions (in contrast, the 4-NTP needs 6 electrons). Halide anions dramatically increased the catalytic activity of the metallic Ag because of the photo-recycling mechanism. As we expected, the halide anion efficacy trended with $I^- > Br^- > Cl^-$.

In contrast, in the presence of only AgX (without metallic Ag), AgCl and AgBr showed no catalytic activity for this reaction (Supplementary Figure 20). AgI has a weak effect on the reduction, but not as efficient as metallic Ag plus I^-. This can be attributed to the much higher photo-sensitivity of AgI compared to other AgXs so that the $[Fe(III)(CN)_6]^{3-}$ can be reduced during the photo-dissociation of AgI (not as a catalyst but as a reducer).

In summary, we tested this recycling mechanism using a different chemical reaction not involving protons, where the halide anion efficacy also trended with $I^- > Br^- > Cl^-$, but AgX alone cannot catalyze this reduction. These results support our proposed mechanism with the photo-recycling Ag-AgX surface acting as the catalyst. Overall, the mechanism is in principle generally applicable.

Supplementary References

1. Talley, C. E. *et al.* Surface-enhanced Raman scattering from individual Au nanoparticles and nanoparticle dimer substrates. *Nano Lett.* **5,** 1569-1574 (2005).

2. Steinigeweg, D. & Schlücker, S. Monodispersity and size control in the synthesis of 20-100 nm quasi-spherical silver nanoparticles by citrate and ascorbic acid reduction in glycerol-water mixtures. *Chem. Commun.* **48,** 8682-8684 (2012).

3. Küstner, B. *et al.* SERS labels for red laser excitation: silica-encapsulated SAMs on tunable gold/silver nanoshells. *Angew. Chem. Int. Ed.* **48,** 1950-1953 (2009).

4. Xie, W., Herrmann, C., Kömpe, K., Haase, M. & Schlücker, S. Synthesis of bifunctional Au/Pt/Au core/shell nanoraspberries for in situ SERS monitoring of platinum-catalyzed reactions. *J. Am. Chem. Soc.* **133,** 19302-19305 (2011).

5. Joseph, V. *et al.* Characterizing the kinetics of nanoparticle-catalyzed reactions by surface-enhanced raman scattering. *Angew. Chem. Int. Ed.* **51,** 7592-7596 (2012).

6. Xie, W., Walkenfort, B. & Schlücker, S. Label-free SERS monitoring of chemical reactions catalyzed by small gold nanoparticles using 3D plasmonic superstructures. *J. Am. Chem. Soc.* **135,** 1657-1660 (2013).

7. van Schrojenstein Lantman, E. M. *et al.* Catalytic processes monitored at the nanoscale with tip-enhanced Raman spectroscopy. *Nat. Nanotechnol.* **7,** 583-586 (2012).

8. Kang, L. L. *et al.* Laser wavelength- and power-dependent plasmon-driven chemical reactions monitored using single particle surface enhanced Raman spectroscopy. *Chem. Commun.* **49,** 3389-3391 (2013).

9. Zhao, L. B. *et al.* Theoretical study of plasmon-enhanced surface catalytic coupling reactions of aromatic amines and nitro compounds. *J. Phys. Chem. Lett.* **5,** 1259-1266 (2014).

10. Huang, J. F. *et al.* Site-specific growth of Au-Pd alloy horns on Au nanorods: a platform for highly sensitive monitoring of catalytic reactions by surface-enhanced Raman spectroscopy. *J. Am. Chem. Soc.* **135**, 8552-8561 (2013).
